# Ziemssen’s Cyclopædia, Vol. IV

**Published:** 1876-11

**Authors:** 


*Cyclopædia of the Practice of Medicine.* Edited by DR. H. VON ZIEMSSEN. Vol. IV. Diseases of the Respiratory Organs. New York, Wm. Wood & Co., 1876.

The fifth volume of this work, already noticed, (Vol. XV. p. 277,) is devoted to the same general subject as this, and Dr. Buck informs the readers of the work that additional matter upon diseases of the respiratory organs excluded from this volume for want of space, will be found at the end of Vol. VII. Article first of this volume upon diseases of the nose, naso-pharyngeal space, pharynx and larynx, is by Dr. Fraenkel. Preceding the description of the diseases of these localities, is an account of the methods of examination, the instruments employed and formulæ for treatment.

The article upon Croup and Diphtheria is by Dr. Steiner, the author of a work upon Children's diseases, which has been recently translated into English. He regards these two affectionr as one disease under dissimilar forms. The only difference, he says, consists in the fact that in croup the exudation takes place upon the free surface of the mucous membrane, while in diphtheria it occurs at the same time within the tissues. In pleuritis much attention is paid to operative treatment. The volume, taken in connection with volume five, forms a very valuable treatise upon diseases of the respiratory system. It is fully abreast with the times, and while there are some things in the line of treatment with which American physicians cannot coincide, there is nothing which can harm them to know.

